# Renewable energy conversion using nano- and microstructured materials

**DOI:** 10.3762/bjnano.10.76

**Published:** 2019-03-26

**Authors:** Harry Mönig, Martina Schmid

**Affiliations:** 1 Physikalisches Institut, Westfälische Wilhelms-Universität Münster, Wilhelm-Klemm-Str. 10, D-48149 Münster, Germany; 2 Fakultät für Physik und CENIDE, Universität Duisburg-Essen, Lotharstr. 1, D-47057 Duisburg, Germany

**Keywords:** energy conversion, energy storage, light harvesting, renewable energy, solar cells

The imposing environmental and economic challenges due to climate change have become a major topic of discussion on the global political agenda. Effectively reducing greenhouse gases in the atmosphere and decreasing air pollution in metropolitan areas requires a paradigm shift away from the conventional practise of fossil fuel combustion. Therefore, the development of new technologies based on renewable energy conversion should be one of the major goals of the global society. The *Beilstein Journal of Nanotechnology* thematic issue “Nano- and microstructures for energy conversion: materials and devices” provides insights into the latest developments in the related fields. Besides a focus on solar-cell concepts [1–5], it also addresses light harvesting by solar fuel production [6–7], and energy storage by batteries [8].

Nanostructured materials can be synthesized by a huge variety of approaches, extending from self-assembled structures [9], over various lithographic techniques [10] and imprinting methods [11], to different crystallization routes [12]. The thematic issue “Nano- and microstructures for energy conversion: materials and devices” covers the photo-electrochemical growth of platinum catalysts at plasmonic hot spots [6], the laser-assisted local growth of chalcopyrite absorbers [4], the preferential reactive ion etching of silicon by morphological anisotropies [5], the oxidation of copper nanoparticles resulting in nanoporous cobalt oxide photocathodes [7], and an approach in which silicon nanoparticles are embedded in an amorphous carbon matrix [8].

In terms of material saving, nano- and microstructured absorbers offer great potential, e.g., via ultrathin absorbers as highlighted for Sb_2_S_3_ hybrid solar cells [1] or via microabsorbers as shown for Cu(In,Ga)Se_2_ [4] in this thematic issue. At the same time, material reduction demands for optical concepts that support efficient collection of the incident solar radiation. In this regard, nanotexturing is of interest, where optical resonances and light scattering can be tailored to give rise to field enhancement, path-length enhancement and finally increased absorption. Nano- and microtextures in Si heterojunction solar cells are addressed from a theoretical point of view in [3], whereas [5] additionally presents an experimental verification of the benefits arising from core–shell nanowire arrays for Si heterojunction solar cells. Contacts with a high surface-to-volume ratio can clearly be seen. Particularly in photovoltaics, they may be prone to increased recombination losses. For other applications, such as water splitting, porous materials may however be desired as the example of Co_3_O_4_ photocathodes in [7] shows.

In general, the nanostructure of any material will strongly affect the corresponding optical and electronic properties by controlling the surface-to-volume ratio and the related morphological characteristics. Besides the concepts for increasing the absorption or the area of a chemically reactive surface, a very established approach concerns bandgap engineering by varying the size and shape of nanoparticles, which enables, for instance, the optimization of the optoelectronic material properties to the solar spectrum [13]. Furthermore, nanostructures can be used to embed sensitive photoactive materials in order to protect them from oxidation or photochemical processes due to exposure to moisture [14].

The mentioned examples of nanoscale materials and device concepts highlight the huge possibilities to tailor the corresponding functional properties in order to optimize the processes involved in energy conversion and storage. Yet, it is important to keep in mind that from an economic point of view, boosting efficiencies alone is not sufficient to establish a certain technology on the market. Rather, efficiencies must be related to the device lifetime and the production cost in order to compete with conventional approaches. It is particularly important whether upscaling by large-area deposition techniques is possible and whether the used precursor substances are available in sufficient quantities at reasonable cost. Therefore, it is highly desirable to use raw materials from cheap earth-abundant elements [15] or to minimize the amount of absorber materials by a combination with optics for efficient light collection [4,16] in order to achieve environmentally friendly production and recycling cycles. This also implies that substances with a high degree of toxicity should be avoided as far as possible [17]. The use of such toxic substances not only leads to a considerable increase in the fabrication cost (due to expensive security measures to meet the health and safety requirements in workspace) but also leads to indirect costs incurred during recycling or disposal of the materials [18–19].

To further establish the various technological approaches using nano- and microstructured materials for energy conversion, several challenges have to be overcome. Besides efficiency, device stability is a major concern, which particularly holds for cases in which organic or hybrid materials are involved [20]. For example protection against device degradation by advanced encapsulation techniques can help to further develop such technologies [21]. As it is the case for conventional materials, interface engineering is also a major factor for device optimization. Due to the significantly increased surface-to-volume ratio of nanostructured materials, the development of interface passivation strategies is one of the major challenges in the case of photovoltaic devices where recombination losses are most harmful at the photoactive interfaces. To a large extent, the difficulties in this endeavour originate from the complexity of interfaces nanostructured in three dimensions [22]. Relating the morphology to results from defect-spectroscopy experiments, theoretical models and device performance could be a valuable approach to comprehensively address such issues.

Despite these challenges, nano- and microstructured materials will certainly play a dominant role in the development of next generation devices for energy conversion. At the same time, the huge variety of devices and material concepts also require massive efforts from the research community together with the related scientific discussions. In this sense, the present thematic issue provides a platform for a contemporary cross-section of topics in this broad field of research.

The *Beilstein Journal of Nanotechnology* is a unique medium for scientific exchange across the traditional disciplines. It is based on a non-profit organization, making it independent from commercial interests, while following the highest standards of open-access publishing. It was a pleasure to work closely with the editorial office and all the authors and reviewers contributing to the present thematic issue.

Harry Mönig and Martina Schmid

Münster and Duisburg, February 2019
